# Effective interventions to improve long-term physiotherapy exercise adherence among patients with lower limb osteoarthritis. A systematic review

**DOI:** 10.1186/s12891-022-05050-0

**Published:** 2022-02-14

**Authors:** Pathmanathan Cinthuja, Nidhya Krishnamoorthy, Gamalendira Shivapatham

**Affiliations:** 1 Department of Allied Health Sciences, Faculty of Medicine, University of Colombo, Colombo, 00800 Sri Lanka; 2 Physio Medicare, Private Physiotherapy clinic, Dehiwala, 10350 Sri Lanka; 3 School of Engineering and Material Science, Queen Mary University of London, Bancroft Road, Mile End, London, E1 4NS UK

**Keywords:** Osteoarthritis, Long term, Exercise adherence

## Abstract

**Introduction:**

Osteoarthritis (OA) is a chronic condition. Physiotherapy is known to be beneficial for people with OA. Patient adherence to physiotherapy exercise is essential for the effective management of OA.

**Objectives:**

To determine different methods used to enhance physiotherapy exercise adherence for a period of more than 12 months among patients with OA and to report the most effective methods to enhance exercise adherence among people with lower limb OA.

**Design:**

Systematic review.

**Methods:**

PubMed, Pedro, Web of Science, and EMBASE databases were searched for randomized controlled trials, cohort studies, case-control studies, and cross-sectional studies published in the English language from 2000 to 2020. The literature search was done on 27 August 2020. Two researchers independently conducted the screening, eligibility assessment, data extraction, methodology quality assessment using the PEDro scale, and risk of bias assessment using RoB2. A narrative synthesis of key outcomes is presented, percentage of adherence rate; Preferred Reporting Items for Systematic Review was used to report the review. Meta-analysis was not performed due to heterogeneity of studies. The study protocol was registered in Prospero (Prospero ID: CRD42020205653).

**Results:**

The primary search strategy identified 5839 potentially relevant articles, of which 5157 remained after discarding duplicates. After screening based on title and abstract, 40 papers were potentially eligible for inclusion. Five of these papers met all predefined eligibility criteria. Introducing methods to enhance exercise adherence has caused a significant increase in exercise adherence for less than 6 or 12 months. There were no significant differences in adherence for more than 12 months with different methods. The results indicate that booster-sessions (89.69%) and telephone-linked communication (86%) had higher percentages for exercise adherence. Secondary outcomes such as pain, stiffness and function show positive outcomes with increasing exercise adherence. However, there were no significant differences on these secondary outcomes.

**Conclusion:**

The booster sessions and telephone-linked communication appear to enhance exercise adherence for more than 12 months among patients with OA. However, the number of high-quality studies is inadequate to confirm our findings. Therefore, more studies with higher methodological quality are needed to determine the best strategies to enhance long-term exercise adherence among people with OA.

**Supplementary Information:**

The online version contains supplementary material available at 10.1186/s12891-022-05050-0.

## Background

Osteoarthritis (OA) is defined as a ‘clinical syndrome of joint pain accompanied by varying degrees of functional limitation and reduced quality of life’ [1]. This is characterized by ‘pathological loss of cartilage, remodelling of adjacent bone and associated inflammation’ [1]. OA can develop as primary osteoarthritis and secondary osteoarthritis where predisposing conditions exist. This is one of the important causes of disability and pain [2]. OA has an impact on individuals, society and the health care system. In terms of individuals, it affects a person’s quality of life by causing pain and reduction in function. OA can affect joints in peripheral either single or multiple joints. Small hand joints, hips and knees are commonly affected joints [1].

OA is managed through non-pharmacological and pharmacological methods. Guidelines have been developed to manage OA. Many guidelines have been published for the management of OA by institutions such as NICE (National Institute for Clinical Excellence), OARSI (Osteoarthritis Research Society International), and EULAR (European League Against Rheumatism). According to the NICE guidelines, education, advice, information access, exercises for strengthening, aerobic fitness and weight loss should be considered as core treatments [1]. Paracetamol and topical NSAIDs should be considered when further treatment is needed [1]. Other options include self-management techniques (local heat and cold, assistive devices), pharmaceutical options and surgery (joint arthroplasty) and non-pharmaceutical treatments (support and braces, shock-absorbing shoes or insoles, TENS and manual therapy), whose efficacy is less well proven, offers less symptom relief and may carry risks for patients [1].

The World Health Organization (WHO) defines ‘the extent to which a person’s behaviour corresponds with agreed recommendations from a healthcare provider’ as adherence [3]. Adherence to guidelines by health care providers and patients is an important factor for the effective management of OA. In patients with osteoarthritis, a low level of adherence to physiotherapy exercise affects the effectiveness and outcomes of prescribed exercise [4–6]. It is important to study effective methods to enhance adherence to core management methods, especially long-term adherence to exercises by OA patient groups.

One of the significant challenges in chronic and long-term conditions is adherence to the exercises or management guidelines. Supervised exercise sessions, refresher sessions, audio or videotapes of exercise programmes, self-management programme and cognitive adherence measures are methods that have been shown to improve exercise adherence [7]. The study findings concluded that supervised and individual exercise therapy and self-management techniques increase exercise adherence among adults with chronic musculoskeletal pain [7]. A Cochrane review suggested self-management techniques, supervised and individualized exercise programmes, activity monitoring, feedback systems, written instructions and behavioural exercise programmes, booster sessions, behavioural graded exercises and peer-delivered programmes have shown adherence potential [3]. A study conducted in Australia to identify the barriers and enablers for the management of osteoarthritis stated that ongoing episodic nature, the availability of visual evidence of joint surface damage on X-ray images and prescribing exercise when it is perceived as a reason for joint surface damage are challenges in the management of OA. This study suggested that clinician adherence to guidelines can be enhanced by decision support and reminder systems, interactive educational strategies and educational outreach and clinical practice audits that provide feedback to clinicians [8].

OA is a chronic condition. It affects people’s mobility and the quality of life. Joint pain, stiffness, decreased range of joint movement, muscle weakness of quadriceps and alterations in proprioception are clinical manifestations of OA [9]. Studies have shown significant benefits of exercise in patients with osteoarthritis, especially exercises focusing on strength, flexibility and aerobic capacity [10]. International guidelines from institutions such as NICE, EULAR and OARSI recommend core exercises and effective management [1, 10], which are key elements of any treatment program for OA [11]. Regular exercise reduces physical impairments and improvements in mobility and mental health [10, 12]. This improves everyday physical function. There are non-pharmaceutical treatments such as support and braces, shock-absorbing shoes or insoles, TENS, and manual therapy; however, these are less well proven in terms of efficacy, offer less symptom relief and carry risks for patients according to NICE guidelines [1]. Long-term clinical success is based on long term adherence to a recommended exercise programme [6, 13]. Life-long exercise is considered crucial for maximizing the well-being and function of adults with knee OA [14]. However, whether patients adhere to these exercises in the long term is not clear [5]. Therefore, it is essential to study adherence to core management methods (especially adherence to physiotherapy exercise by patients with osteoarthritis), identify methods to enhance long-term adherence to these guidelines for more than 12 months, and follow up to monitor or evaluate the level of adherence to guidelines and investigate the effectiveness of the intervention methods to enhance the exercise adherence among patients with osteoarthritis. Many reviews have been conducted to assess exercise adherence for ≤12 months [7, 15]. This study aimed to determine the methods to enhance long term (i.e. more than 12 months) exercise adherence among patients with lower limb osteoarthritis and to identify the most effective methods to enhance exercise adherence among patients with osteoarthritis.

### Methodology

A systematic review was performed to identify effective methods to enhance long-term (> 12 months) exercise adherence among patients with lower limb osteoarthritis. This systematic review was conducted according to the Preferred Reporting Items of Systematic Reviews and Meta-Analyses (PRISMA) guidelines [16]. This protocol was registered (the protocol was not peer reviewed and registration during the 2020 pandemic was automatically published exactly as submitted) in Prospero (Prospero ID: CRD42020205653).

### Search strategy

A literature search was performed using electronic databases on 27 August 2020 (PubMed, Pedro, EMBASE and Web of Science). Databases were searched for randomized controlled trials, cohort studies, case-control studies, and cross-sectional studies published in English from 2000 to 2020. The search strategy included only terms relating to or describing the interventional methods. The keywords used for the search were as follows: osteoarthritis, arthritis, hip, lower limb, knee, extended period, long period, compliance (Table 1), adherence (Table 1), follow-up, engagement (Table 1), strengthening, exercise, physical therapy, rehabilitation therapy, resistance training, muscle strengthening and aerobic exercise. Full search strategy and individual results can be found in electronics supplement. The searches were re-run just before the final analyses and further studies were retrieved for inclusion. The date of data extraction was 4 September 2020. Cited articles among included studies were also checked.

**Table 1:** Operational definition

Term	Operational definition
Compliance	the fact of obeying a particular law or rule, or of acting according to an agreement
Engagement:	the fact of being involved with something:
Adherence:	the act of doing something according to a particular rule, standard, agreement

### Selection criteria

Full papers were included in the final analysis. Randomized controlled trials (RCTs), cohort studies, case-control studies, cross-sectional studies published in English and peer-reviewed journals were eligible for inclusion.

Studies conducted among the population with lower limb knee or hip joint osteoarthritis that recruited both males and females aged above 18 years, carried out physiotherapy exercise as a primary intervention and followed up for more than 12 months were eligible for inclusion. Studies including lower limb muscle pathology or systemic or metabolic or neurological disease were excluded.

Therapeutic exercise is the systematic, planned performance of bodily movements, postures or physical activities intended to provide a patient/client with the means to remediate or prevent impairments; improve, restore or enhance physical function; prevent or reduce health-related risk factors; and optimise overall health status, fitness or sense of well-being [17]. Physical therapists select, prescribe and implement exercise activities when the examination findings, diagnosis and prognosis indicate the use of therapeutic exercise [18, 19]. Therapeutic exercises for osteoarthritis include strengthening, stretching and cardiovascular exercises [20, 21].

### Outcome

Articles were included if the intervention(s) aimed to improve adherence, compliance or engagement with exercise, compared with either no adherence or engagement intervention. A paper with a comparator group that was also undertaking the exercise programme and where a no-intervention control group occurred for a long duration but there were at least two active intervention groups to offer a comparison was also included.

### Data extraction

Two independent researchers retrieved titles and abstracts of studies using the search strategy and those from additional sources, i.e. cited articles among included studies, were screened independently by two reviewers (CP & NK). A pilot test was done on the data extraction sheet and based on that a placeholder section for final results of the studies and methodology quality assessment results were added to the final data extraction form. The full text of these potentially eligible studies was retrieved and independently assessed for eligibility. Disagreements were resolved through discussion with a third reviewer (GS).

A predetermined extraction form was used to extract data from the included studies to assess study quality and evidence synthesis. Study setting, study population, participant demographics, details of the intervention, methods used to enhance exercise adherence and study methodology were extracted independently by two review authors. Discrepancies were identified and discussed between the two researchers (CP & NK) to resolve any differences of opinion, and a third reviewer’s opinion was taken when it was not possible to reach a consensus. Relevant authors were requested to provide missing data; one such attempt by investigators was made via email.

### Quality and risk of Bias assessment

Two reviewers independently conducted methodology quality assessment using the Pedro scale, and the Risk of Bias Assessment tool for randomised control trials, RoB2 [22], was used to assess the risk of bias (Table 2). The PEDro scale is composed of 11 items, of which the first item is only applicable for specification of eligibility criteria, and it was not considered as part of calculating the overall PEDro score. Each item was given one point, and the total score could be between 0 to 10 points. Studies that scored ≥4 points were considered ‘high’ quality and studies that scored < 4 points were considered to be of ‘low’ methodological quality [23]. Studies heterogeneity checked for potential meta-analysis. RoB2 focuses on different aspects of trail design, conduct and reporting. Judgment about risk of bias arising from each domain is generated by an algorithm, based on the questions. Domains include bias arising from the randomization process, bias arising from the timing of identification or recruitment of participants, bias due to deviation from the intended interventions (effect of assignment to intervention), bias due to deviations from the intended interventions (effect of adhering to intervention), bias due to missing outcome data, bias due to measurement of the outcome and bias in selection of the report result. Based on the assessment, a domain can be ‘low’ or ‘high’ risk of bias or it can be expressed as ‘some concerns’ [24].

**Table 2:**
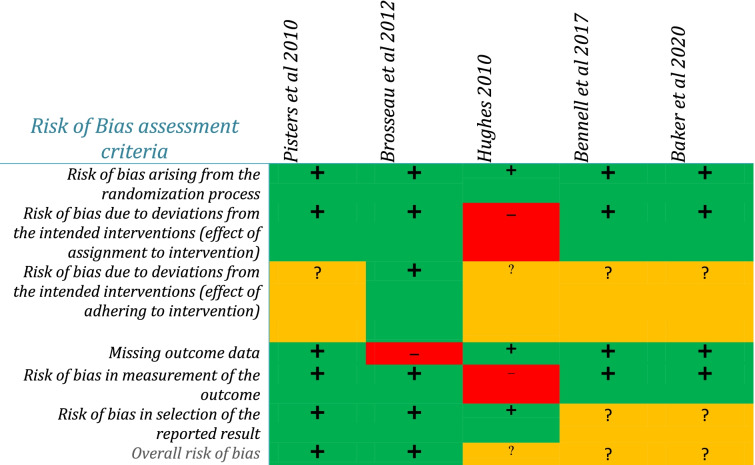
The table shows risk of bias assessment using Risk of Bias Assessment tool for systematic reviews tool

Low risk of bias (green), high risk of bias (red), unclear risk of bias (amber)

Cohen’s Kappa analysis was performed to identify the agreement between raters on the methodology quality of the included studies. The percentage of agreement on methodological quality was noted as 80%. Moderate interrater reliability with k = 0.72, SE = 0.21 [25], was analysed using SPSS (statistics version 26.0.0; SPSS Inc., Chicago, IL).

## Results

### Selection of studies

**Fig. 1:**
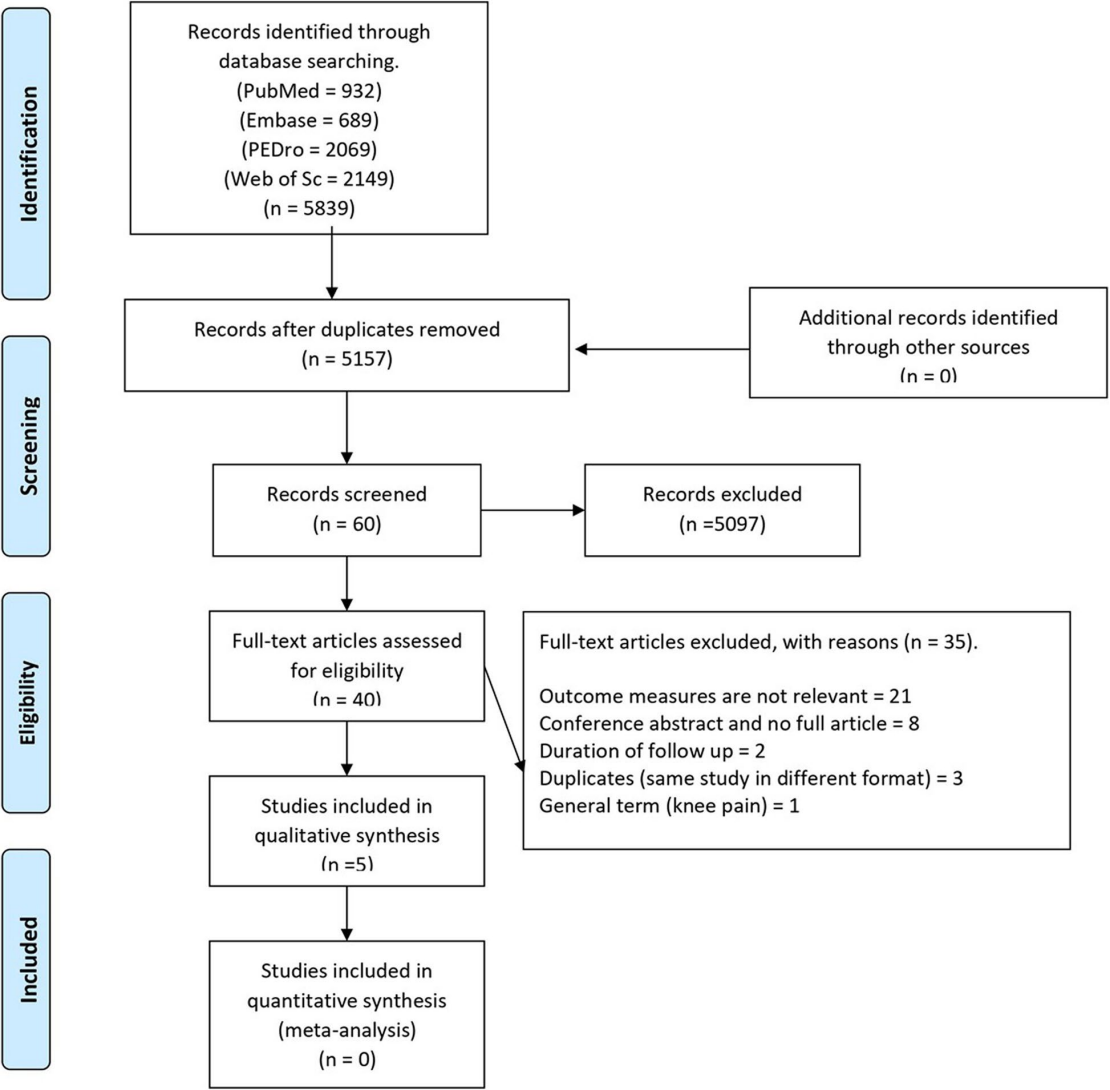
PRISMA Flow chart

The primary search strategy identified 5839 potentially relevant articles of which 5157 remained after discarding duplicates (Fig. 1). The date of data extraction was 4 September 2020. After screening based on title and abstract, 40 papers were potentially eligible for inclusion. The number of additional articles from external searches was zero. Five of these papers met all the predefined eligibility criteria.

Studies were excluded due to many reasons, the most common reason being the follow-up duration. A follow up of more than 12 months was not conducted for most of the trials. Studies were also excluded when physiotherapy exercise was not the intervention for patients with osteoarthritis or there was no intervention to improve exercise adherence.

### Characteristics of included studies

In total, 1113 patients with lower limb osteoarthritis were included in the five studies that were analysed. All studies used an RCT design and included patients who were currently diagnosed with knee osteoarthritis, hip osteoarthritis or both. The median sample size was 223 participants (range = 104–419). The mean age was 65.37 (± 3.42) years. The mean length of the follow-up was 26.6 months (range = 18–55 months). Of the five studies, four reported using the intention-to-treat (ITT) method for missing data, and one study did not confirm the type of analysis used. In the selected five studies, booster sessions, the behavioural approach, telephone sessions, motivation calls and telephone reinforcement were used to enhance exercise adherence.

In the five studies, mean duration of exercise interventions was 19.6 weeks (range = 6–48 weeks). Interventions include individually-tailored exercises with complete protocols including written materials such as education messages, activity diaries, performance charts [6], walking sessions [26], 60 min of stretching/flexibility, low-impact aerobics, strengthening and balance exercises followed by 30 min of manual-based, group problem-solving/health education for managing OA with PA [27], 30-min consultations with a physiotherapist over six months for education, home exercise and physical activity advice [28] and six-week group exercise classes [29].

In the five studies, the behavioural approach at a community-based walking club [26], telephone sessions with a health coach [28], TLC motivational calls [29], telephone reinforcement [27] and booster sessions [6] were the strategies used to enhance exercise adherence among participants in the experimental groups. The adherence rate for the five-booster session group was 89.69, 59.4% for the behavioural approach group, 39% for 6–12 telephone sessions with a health coach and 86% for the telephone-linked communication group. Individual scores were not available for the telephone reinforcement group.

In addition to exercise adherence, physical activity level, lower extremity pain, stiffness, function, sit-stand, a six-minute distance walk, anxiety, or depression, the Western Ontario and McMaster Universities Arthritis Index (WOMAC) pain, WOMAC physical activity, function subscale, functional performance assessment, quadriceps and hamstring strength were other outcomes measured in these studies.

### Assessment of methodological quality and quantitative analysis

The five included studies were scored using the PEDro scale. Methodological quality ranged from 3 to 8 as rated on the PEDro scale with a median score of 6.8. The methodology quality assessment is summarized in Table 3. All studies were rated as high qualitative studies with a score of ≥4 [23], of which 2 scored 8/10 points and 2 scored 7/10 points. Quantitative analysis by pooling outcome data (meta-analysis) or a best-evidence synthesis was inappropriate. This is due to the incomparability of outcome data caused by the heterogeneity of techniques to enhance exercise adherence.

**Table 3:** Table shows methodological quality assessment of included studies assessed using Pedro scale

Methodology quality assessment	Pisters et al 2010 [6]	Brosseau et al 2012 [23]	Hughes et al 2010 [27]	Bennell et al 2017 [25]	Baker et al 2020 [26]
Eligibility criteria were specified (Not used in score generation)	Y	Y	Y	Y	Y
Subjects were randomly allocated to groups	Y	Y	Y	Y	Y
Allocation was concealed	Y	Y	NG	Y	Y
Groups were similar at baseline	Y	Y	Y	Y	NG
Subjects were blinded	Y	N	N	Y	NG
Therapist who administered the treatment were blinded	N	N	N	N	N
Assessors were blinded	Y	Y	N	Y	Y
Measures of key outcomes were obtained from more than 85% of subjects	N	N	N	N	Y
Data were analysed by intention to treat	Y	Y	NG	Y	Y
Statistical comparisons between groups were conducted	Y	Y	Y	Y	Y
Points measures and measures of variability were provided	Y	Y	NG	Y	Y
Total score	8	7	3	8	7

*Y* Yes, *N* No, *NG* Not given

Table 4 summarizes the characteristics of the studies included in the analysis. The domains commonly lacking in the included studies were as follows: the therapists who administered the treatment were blinded, measures of key outcomes were obtained from more than 85% of subjects and subjects were blinded.

**Table 4:** Table shows the data extraction of included five studies

	Pisters et al. 2010 [6]	Brosseau et al. 2012 [3]	Bennell et al. 2017 [25]	Baker et al. 2020 [26]	Hughes et al. 2010 [27]
Design	Single-blind cluster-randomised trial	Single-blind, randomised control trial	Randomised control trial	Single-Blind, parallel-arm randomised controlled trial	Randomised control trial
Sample size	200 (Exp (*n*) = 97, Con (*n*) = 103)	222 [W = 79, WB = 69, Con = 74]	168	104 [Con = 44, TLC = 45]	419 [Negotiated TR = 103, Negotiated No TR = 98, mainstream Tel = 105, Main No Tel = 113]
Duration- Follow up (months)	up to 55	18 (12 intervention, 6 month follow up)	18	24	18
Population([N], Gender, Age, Joint involved)	Exp [Age = 65 [7], Gender (males) = 24(25)], Con [Age = 65(8), Gender (males) = 22 (21)]	Age [w = 63.9(10.3), WB = 63.9 (8.2), Self-directed control = 62.3(8.6)], Men/Women, (%) [w = 24(30.4)/55(69.9), 18(26.1)/51(73.9), 69(31.1)/153(68.9)	Age [PT + Coaching = 61.1 ± 6.9, PT = 63.4 ± 7.8], Male, n (%) = [PT + Coaching = 27(32), PT = 35(42)]	Age [TLC = 65.8 ± 6.6, Con = 64.5 ± 8.3], female n (%) [TLC = 42 (80.8), Con = 43(82.7)]	Majority female, Age 71.1
Study population	200 Hip and/or knee OA patients	222 patients with mild to moderate OA	168 inactive adults ≥50 years with knee pain on a numeric rating scale ≥4 and knee OA	104 knee OA patients	419 Community dwelling older adults with LE OA
Exercise intervention (Type, frequency, duration, intensity)	Maximum if 18 sessions over a 12-week period. The complete protocol included written materials such as education messages, activity diaries, performance charts.	Walking programme (supervised walking programme or unsupervised/self-directed walking programme)	5* Individual Physiotherapy sessions	6-Week group exercise class and monthly Automated Phone messages to strength Train and Complete Exercise Logs	Fit and strong programme
Adherence facilitation	Five booster sessions in week 18,25,34,42 and 55.	Behavioural approach at the community-based walking club	6 to 12 telephone sessions with a health coach	TLC motivational calls	Telephone reinforcement
Outcome measure(s)	Participants self-rated adherence, SQUASH	Validated questionnaire, Physical tests	Self-report questionnaire, 11-point NRS, WOMAC, NRS pain on walking, WOMAC pain scale, Assessment of QoL, Physical activity for the elderly (PASE), AAS, Accelerometer-based device	Single self-report item, WOMAC pain, Physical function subscales, Biodex System 3	Physical activity Maintenance, WOMAC, functional lower extremity strength (timed-stand), functional exercise capacity (6-min distance walk), Body Mass Index, Depression
Lost to follow up	21 (Exp (n) = 10, Con (n) = 11), 20% loss to follow up	18 months [W = 44.3%, WB = 40.6%, Con = 52.1%]	Loss to follow up 26 of 168 (15%), 32 of 168 (19%) and 40 of 168 (24%)	out of 52, [TLC = 7/52, Con = 8/52]	91 unable to locate, 29 unable to schedule, and 40 refused
Adherence rate	–	–	PT + Coaching 3.8 [95% CI-3.1, 4.6] versus PT 3.6 [95% CI 2.9, 4.4], mean difference 0.2 [95% CI − 0.8, 1.2]	[Mean control group = 4.01 [95% CI 3.03,4.99, Mean for TLC 3.63 [95% CI 2.70, 4.56]; *P* = 0.57)	74% the participants completed measurement at 12 months, 62% (259) at 18 months
Long term outcome	Significant difference is present. Higher in experimental group	There is difference. But significant level is not mentioned	No significant difference between groups	No significant difference between groups	TR positively affect perceptions around engagement

*Exp* Experimental group, *Con* Control group, *W* Walking, *WB* Walking and Behaviour, *TLC* Telephone-Linked Communication, *TR* Telephone Reinforcement, *Tel* telephone, *QoL* Quality of Life

### Measurement instruments and outcome measures of adherence

Adherence to exercise intervention was measured using a questionnaire [6], the number of attended walking sessions divided by the number of prescribed sessions recorded by the exercise therapist and participants’ completed logbooks [26], self-report questionnaires completed at home at baseline, 6, 12 and 18 months [28], single self-report item interviewer-administered at 12 and 24 months and administered by phone at 18 months [29] and self-administrated questionnaires [30]. Out of these five studies, four have reported adherence as one of the primary outcomes [6, 26, 29, 30].

## Discussion

This review summarizes different methods to enhance exercise adherence among people with knee osteoarthritis for more than 12 months. Different factors were reported for loss of follow-up with the exercise programme. This section will focus on the main findings related to the study.

### Summary of main findings

Five studies were eligible according to the inclusion criteria. Strategies used to enhance exercise adherence vary among trials. Booster sessions, behavioural approaches, telephone sessions, telephone linked communication, motivational calls and telephone reinforcement were used as strategies in these studies.

The long-term exercise adherence rate of the following methods varied between studies. According to the results, booster sessions and telephone-linked communication had higher percentages for exercise adherence. The experimental group who received booster sessions had a maximum of 18 sessions (individually-tailored exercises, education message, activity diaries and performance chart) over a 12-week period, followed by five booster sessions in weeks 18, 25, 34, 42, and 55 [6]; the experimental group who received telephone linked communication received five 30-min consultations with a physiotherapist over 6 months for education, home exercise, physical activity advice and 6–12 telephone coaching sessions by a clinician trained in behavioural change support for exercise and physical activity [28]. Previous studies suggested behavioural strategies and educational approaches will improve exercise adherence [15, 31]. Exercise adherence is influenced by different factors. These can be divided into intrinsic/personal factors or extrinsic factors. Health care and well-informed motivated health care providers, lifestyle-related issues and the extent of ongoing social or therapist contacts, knowledge about their exercise regimen and its potential effectiveness and degree of self-motivation are some of the factors related to exercise adherence [15]. During booster sessions and telephone linked communication, some of these factors are addressed, mainly behavioural change and knowledge and ongoing therapist contacts, which increase exercise adherence. Therefore, future studies should investigate the effectiveness of these two strategies in enhancing exercise adherence.

According to the systematic review results, introducing methods to enhance exercise adherence has only a short-term impact. There are no significant differences in long-term adherence with different methods. Further, most of the secondary outcomes show positive outcomes with increasing exercise adherence. However, there are no significant differences in outcomes between the experimental and control groups. The non-significant difference might be due to a lack of adherence to the exercise guidelines. In the future, it is recommended that an individual patient should be assessed and the potential reasons for lack of adherence; for example, lack of motivation, socioeconomic situation, family and work commitments, should be assessed when formulating exercise adherence strategies for an individual. This might potentially enhance their exercise adherence.

Outcome measures were used to measure exercise adherence in the selected studies. Most of the studies used a self-rated adherence rate. In previous reviews similar methods were used to measure adherence rate [15]. Self-rated adherence rate might have a bias as it depends on a participant’s response. There is a possibility for overestimation of exercise through recall or social desirability bias [15]. Therefore, a standard method, such as attendance records and validated wearable activity trackers, should be used in future studies to report exercise adherence rates [23].

Commonly reported reasons for loss of follow up were co-morbidity, selected surgical intervention option, family circumstances, decline to participate, lack of motivation, pain increase, loss of contact, withdrawal from the study due to personal reasons, other illness, family illness, lack of time, change in location and death. It is reported that the barriers to continuing with the interventions were weather conditions and health problems of participants’ partners. A study was conducted to determine the predictors of adherence to exercise interventions during and after cancer treatment. According to the results, prominent predictors of exercise adherence were the location of the rehabilitation centre and motivation for exercise [23]. These factors are similar to people with osteoarthritis. Studies among people with OA indicated that pain, mental health, body mass index, important problems associated with disabling knee osteoarthritis, the extent to which patients are compensated for their disability, prior exercise behaviours, exercise beliefs and knowledge and lack of time are associated with exercise adherence [14, 32–34]. Therefore, it is important to consider these barriers and reasons when identifying interventions to enhance exercise adherence among people with long-term conditions.

This review finding is consistent with previous studies. The existing literature suggests that there is a significant difference in short-term exercise adherence but then patients fail to maintain adherence in long term [15, 33]. It is mentioned that it is difficult to determine which component of the intervention was truly having a positive effect on adherence [34]. These findings are similar to this study’s findings. Further, the literature suggests that there are different determinants [14] for exercise adherence that include both intrinsic and extrinsic factors, and there is no single strategy that can be effective in promoting adherence among people with arthritis [14]. The studies included in the current review used types of intervention that addressed different intrinsic and extrinsic factors. All included studies addressed either one of the intrinsic/extrinsic factors or both factors. Lack of adherence to exercise is the main reason for decline in benefits over time and their disappearance in the long term. Therefore, there is a limited difference in long-term follow up results of these studies. In this review, it appears that mainly behavioural change, awareness about their condition and ongoing therapist contacts have an influence on increasing exercise adherence. Moreover, it should be noted that behavioural patterns are more likely to differ for a larger population. Therefore, it is important to consider that fact when formulating an intervention strategy.

### Strengths

One of the strengths of this systematic review is that four studies were of ‘high’ methodological quality. This systematic review used the PRISMA guidelines for reporting systematic review and the PEDro scale.

### Limitations

Several limitations should be considered during this study’s interpretation and be addressed in future research. First, only five studies that assessed exercise adherence for more than 12 months were included in this systematic review. Different exercise interventions and exercise adherence strategies were used in these five studies with variations in duration and intensity. The heterogeneity is noted in these studies. Therefore, quantitative analysis by pooling outcome data (meta-analysis) or a best evidence synthesis was inappropriate. The interrater reliability is noted as 0.72 because of the small number of studies included in this study.

In the existing literature, only a limited number of studies focused on exercise adherence for more than 12 months. Also, self-reported exercise adherence was used to assess the adherence rate. Exercises should be followed as one of their routine activities among people with chronic conditions such as osteoarthritis. Many studies reported exercise adherence for a period of less than 12 months or as long as patients actively participate in clinical visits. However, follow up should continue for a longer duration to ensure that patients achieve the benefits of the prescribed exercise programme. These limitations should be addressed in future studies.

The study only included articles published in English. An extensive database review was conducted using a broad search strategy. However, there is a possibility for publication bias and selective reporting biases.

### Future research

Only five studies were eligible according to the criteria. Therefore, in the future, more studies should be conducted to identify the best strategies to maintain exercise adherence for more than 12 months among people with osteoarthritis. In addition, attendance records and validated wearable activity trackers should be used to calculate exercise adherence among participants.

According to the present study, booster sessions and telephone linked communication showed a higher adherence rate. It appears that mainly behavioural change strategies and awareness about their condition and ongoing therapist contacts have an influence on increasing exercise adherence. It is suggested that future studies investigate further these two techniques for long term exercise adherence among OA patients or in any chronic conditions that require continuous exercise adherence.

In this review, articles published in English language were included. In the future, publications in all languages should be considered.

### Clinical implications

This review findings suggest that behavioural changes strategies, patient awareness about their condition and continuous therapist contacts have a positive influence on long-term exercise adherence. Therefore, clinicians should incorporate behavioural changes strategies in their management in addition to exercise prescription. Clinician should educate their patients about the nature of OA and the importance of long-term exercise adherence to achieve good outcomes from exercise programme.

Success of rehabilitation protocol implies on long term adherence of exercise programme, and this review suggests that having a periodical contact with person with OA would provide massive clinical benefits.

## Conclusion

According to the results, booster sessions and telephone-linked communication had higher percentages for exercise adherence. Results indicated that utilizing different strategies does not have a significant influence on the exercise adherence a period of more than 12 months and a non-significant difference in the primary and secondary outcomes. However, the number of high-quality studies is inadequate to confirm our findings. Therefore, high quality future studies are needed to determine the best strategy to enhance long-term exercise adherence among patients with osteoarthritis.

## Supplementary Information

**Additional file 1.**

## Data Availability

Supplementary file is attached with search strategies. The datasets used and/or analyzed during the current study are available from the corresponding author [CP] on reasonable request.

## Abbreviations

OA: Osteoarthritis
NICE: National Institute for Clinical Excellence
OARSI: Osteoarthritis Research Society International
EULAR: European League Against Rheumatism
WHO: World Health Organization
PRISMA: Preferred Reporting Items of Systematic Reviews and Meta-Analyses
ITT: Intention-To-Treat
TLC: Telephone linked Communication
WOMAC: Western Ontario and McMaster Universities Arthritis Index
Exp: Experimental group
Con: Control group
W: Walking
WB: Walking and Behaviour
TR: Telephone reinforcement
Tel: Telephone
QoL: Quality of Life
