# Supplementary material for: Effective interventions to improve long-term physiotherapy exercise adherence among patients with lower limb osteoarthritis. A systematic review
Source: BMC Musculoskelet Disord. 2022 Feb 14;23:147. doi: 10.1186/s12891-022-05050-0 (PMC8842523; doi:10.1186/s12891-022-05050-0)
Supplement: Supplementary file 1 — Additional file 1. [file 12891_2022_5050_MOESM1_ESM.docx]

EMBASE

- (knee:ti,ab,kw OR hip:ti,ab,kw OR 'knee joint':ti,ab,kw OR 'hip joint':ti,ab,kw OR 'lower limb':ti,ab,kw OR leg:ti,ab,kw OR forelimb:ti,ab,kw)
- AND
- ('knee osteoarthritis':ti,ab,kw OR 'hip osteoarthritis':ti,ab,kw OR osteoarthritis:ti,ab,kw OR arthritis:ti,ab,kw)
- AND
- ('long term':ti,ab,kw OR 'long time':ti,ab,kw OR 'long period':ti,ab,kw OR 'extended period':ti,ab,kw OR 'extended time':ti,ab,kw OR 'extended term':ti,ab,kw OR 'compliance period':ti,ab,kw OR 'recovery time':ti,ab,kw OR 'recovery period':ti,ab,kw OR 'recovery term':ti,ab,kw OR adherence:ti,ab,kw OR 'follow* time':ti,ab,kw OR 'follow* duration':ti,ab,kw OR duration:ti,ab,kw)
- AND
- (physio*:ti,ab,kw OR strength*:ti,ab,kw OR exercise*:ti,ab,kw OR 'physical therapy':ti,ab,kw OR 'rehabilitation therapy':ti,ab,kw OR 'resistance train*':ti,ab,kw OR 'muscle strength*':ti,ab,kw OR therap*:ti,ab,kw OR 'treatment protocol':ti,ab,kw OR 'treatment method*':ti,ab,kw OR 'treatment type':ti,ab,kw)
- AND
- ([controlled clinical trial]/lim OR [randomized controlled trial]/lim) AND [2000-2020]/py

Web of Science

- **OPIC:** (knee  OR hip  OR “knee joint”  OR “hip joint”  OR “lower limb”  OR leg  OR foreleg)
- *AND*
- **TOPIC:** (“knee osteoarthritis”  OR “hip osteoarthritis”  OR osteoarthritis  OR arthritis)
- *AND*
- **TOPIC:** (“long term”  OR “long time”  OR “long period”  OR “extended period”  OR “extended time”  OR “extended term”  OR “compliance period”  OR “recovery time”  OR “recovery period”  OR “recovery term”  OR adherence  OR “follow* time”  OR “follow* duration”  OR duration)
- *AND*
- **TOPIC:** (physio*  OR strength*  OR exercise*  OR “physical therapy”  OR “rehabilitation therapy”  OR “resistance train*”  OR “muscle strength*”  OR therap*  OR “treatment protocol”  OR “treatment method*”  OR “treatment type”)
- **Refined by:** **DOCUMENT TYPES:** ( ARTICLE OR PROCEEDINGS PAPER ) AND
- **LANGUAGES:** ( ENGLISH )
- **Timespan:** 2000-2020. **Indexes:** SCI-EXPANDED, SSCI, A&HCI, CPCI-S, CPCI-SSH, ESCI.

Pubmed

- ((knee[Title/Abstract] OR hip[Title/Abstract] OR "knee joint"[Title/Abstract] OR "hip joint"[Title/Abstract] OR "lower limb"[Title/Abstract] OR leg[Title/Abstract] OR forelimb[Title/Abstract] OR "knee osteoarthritis"[Title/Abstract] OR "hip osteoarthritis"[Title/Abstract] OR osteoarthritis[Title/Abstract] OR arthritis[Title/Abstract])
- AND
- ("long term"[Title/Abstract] OR "long time"[Title/Abstract] OR "long period"[Title/Abstract] OR "extended period"[Title/Abstract] OR "extended time"[Title/Abstract] OR "extended term"[Title/Abstract] OR "compliance period"[Title/Abstract] OR "recovery time"[Title/Abstract] OR "recovery period"[Title/Abstract] OR "recovery term"[Title/Abstract] OR adherence[Title/Abstract] OR "follow* time"[Title/Abstract] OR "follow* duration"[Title/Abstract] OR duration[Title/Abstract]))
- AND
- (physio*[Title/Abstract] OR strength*[Title/Abstract] OR exercise*[Title/Abstract] OR "physical therapy"[Title/Abstract] OR "rehabilitation therapy"[Title/Abstract] OR "resistance train*"[Title/Abstract] OR "muscle strength*"[Title/Abstract] OR therap*[Title/Abstract] OR "treatment protocol"[Title/Abstract] OR "treatment method*"[Title/Abstract] OR "treatment type"[Title/Abstract])
